# Benthic fauna on the edge between different seas—signs of climate change in the Sound (Öresund)?

**DOI:** 10.7717/peerj.20996

**Published:** 2026-04-24

**Authors:** Peter Göransson

**Affiliations:** The Sound Fund, Helsingborg, Sweden

**Keywords:** The Sound, Öresund, Benthos, Benthic fauna, Macrofauna, Distribution shifts, Temperature, Hypoxia, Haploops, Climate change

## Abstract

The major goal of this study was to identify long term (1998–2022) changes within the macrobenthic communities in the Sound (Öresund), with special emphasis on climate change. Bottom trawling in this area has been banned since 1932. This was compared to changes of the same species at the surrounding Swedish west coast in the north (the Kattegat and the Skagerrak) and the Baltic Sea in the south. Results are also related far back in time in the Sound as the benthic fauna has been unusually well-studied since the 1800s. It is of special interest to study the faunal response to the rise in bottom water temperature which has been recorded in later years. The Sound between Denmark and Sweden is situated between the Baltic Sea, one of the largest brackish waters, and the Kattegat, an extension of the Atlantic. This location provides special conditions for a benthic fauna with a northern touch. Brackish water species typical of the Baltic occur here, but also marine cold-water species typical of the Arctic. Many species therefore live on their edge of distribution in the Sound. The development in abundance 1998–2022 in the Sound with its trawl-ban was similar to heavily trawled areas along the Swedish west coast. Species with a northern distribution were found to decrease parallel with an increase in species with a southern distribution. The sharper reduction in abundance in the Sound compared to surrounding seas may be due to the fact that many species here live at the extreme edge of their range. Indications of disturbed reproduction and submergence in some northern species were also recorded. The most likely explanation of these changes is the increase in temperature which affects many processes that may act in synergy. The transition from a *Haploops* community to an *Amphiura* community probably started with hypoxia and high temperatures. Also, temperature and decreasing levels of nitrogen, which control primary production, may have created relative food shortages as *Haploops* especially seems to benefit from a high trophic level. The change in faunal composition implied a reduction in gamma diversity but also a loss in nutritional value for commercial fish. Increased temperature is likely an explanation why northern species with non-pelagic larvae have decreased and why southern species with pelagic larvae have increased in recent years. This study, where comparisons are difficult because of many complicating factors, verifies that broken time series is an urgent problem for long-term ecological and environmental studies. It is important for the future to preserve long-term series of data collected at the same location and with the same methods.

## Introduction

### The aim of this study

The major goal of this study was to identify long term (1998–2022) changes within the macrobenthic communities in the Sound (Öresund), with special emphasis on climate change. This was compared to changes of the same species at the surrounding Swedish west coast in the north (the Kattegat and the Skagerrak) and the Baltic Sea in the south. This is especially important as a rise in bottom water temperature has been recorded in later years. The study is also a follow-up and a wider geographical perspective on a similar study in the adjacent Kattegat 1993–2016 (Göransson, 2017a). Assessing long-term changes in the benthic fauna of the Sound in response to warming and comparing them with neighbouring areas represents a significant scientific gap, especially given the region’s unique nature, marked by the historical ban on trawling and its status as a transition zone between different environmental regimes.

### Background

The Sound (Öresund) between Sweden and Denmark is situated between the Baltic Sea in the south, one of the largest brackish waters on earth, and the Kattegat in the north, an extension of the Atlantic. Both brackish water species and glacial relics, typical for the Baltic Sea occur here, as well as marine species, among them several typical for the Arctic region. Many species therefore live on their edge of distribution in the Sound. Range edges are where colonization and extirpation processes unfold (Fredston et al., 2021).

There are relatively few reports of changes of species distribution related to climate change (Poloczanska et al., 2016). Large changes in benthic macrofauna have been predicted (Thomas, Cameron & Green, 2004; Hiscock et al., 2004; Reiss et al., 2011; Birchenough et al., 2015). However, only relatively minor changes have been reported (Barry et al., 1995; Southward et al., 2004; Mieszkowska et al., 2006; Beukema, Dekker & Jansen, 2009; Hiddink, Burrows & Molinos, 2015). Variations in the North Atlantic Oscillation climate index are often associated with changes in the benthic fauna (Tunberg & Nelson, 1998; Kröncke et al., 2019; Kröncke et al., 2011).

Tropical and high-latitude species are considered particularly vulnerable to warming (Tewksbury, Huey & Deutsch, 2008). However, Rousi et al. (2013) highlights increased temperature as an explanatory variable for a dramatic shift in species composition in the northern Baltic in the 1990s. Göransson (2017a) connected increased temperature in the bottom water to a decrease in northern species in the Kattegat 1993–2016. Hale et al. (2017) reported northward distribution shifts along the US Atlantic Coast. Gaudin et al. (2018) reported observed decreases in the occurrence of most cold-water species and increases of most warm-water species in the English Channel in connection with increased bottom temperature since 1960. Pinsky, Selden & Kitchel (2020) emphasizes that the geographic distributions of marine species are changing rapidly, with leading range edges following climate poleward, deeper, and in other directions and trailing range edges often contracting in similar directions. However, Fredston et al. (2021) suggests that range edges of temperate North American marine species have largely maintained the same edge thermal niche during periods of rapid change.

### Study area

The meeting of the brackish water sea and the world ocean creates a uniquely strong stratification of the water column. The Limhamn/Drogden sill in the southern Sound, at only 8 m depth, forms the natural boundary between the brackish Baltic Sea and the saltier Atlantic extension (Skagerrak, Kattegat, and the Sound). The meeting of these waters gives rise to a rare dramatic and dynamic hydrography. The prevailing conditions are a strong Baltic northward surface current down to about 10 m depth (10–15 PSU), below a very strong halocline down to 20 m depth (15–30 PSU) and at bottom a southward salty bottom current (>30 PSU). The deepest bottoms are sometimes exposed to hypoxia because the extremely strong halocline prevents exchange with the oxygen-rich surface water.

There is an increasing gradient in salinity from the south in the Sound towards the north on the west coast, mainly in the surface water, but the deep bottom water (>25 m) is relatively stable above 30′. Conversely there is a decreasing gradient in salinity from the Sound into the Baltic Sea with near limnic conditions in the Bothnian Bay. The temperature also varies considerably between areas. Especially the much cooler Baltic Sea differs from the Sound and the west coast. There is also an increasing gradient in temperature from the Sound to the north on the west coast.

The recruitment of salinity-demanding benthic animals with pelagic larval stage is largely dependent on the transport of larvae from the adjacent Kattegat to the north. This is due to the low salinity of the strait’s surface water (Thorson, 1946). The bottom of the Sound also serves as a trap for phytoplankton from the neighboring Kattegat which could explain the comparatively large biomass of benthic invertebrates in the northern Sound (Nicolaisen & Christensen, 1986). Bottom trawling fishery on Norway lobster *Nephrops norvegicus* is intensive on the silty-clay bottoms deeper than 30 m in the central Kattegat and impact the benthic fauna in the Kattegat and therefore a decrease in the transport of pelagic larvae to the Sound (Hansen & Andersen, 2024). However, benthic species with a short pelagic larval stage or non-pelagic larvae are recruited internally in the Sound (Thorson, 1946).

### The northern touch of the fauna

The special hydrographic conditions create the prerequisites for a diverse benthic macrofauna with a high share of Arctic-Boreal species in the Sound. Studies of the fauna have been ongoing since the early 1800s (Örsted, 1844; Cedhagen & Christiansen, 2025), but Einar Lönnberg was probably the first to relate the marine species to zoogeography (Lönnberg, 1898). He considered that the benthic fauna had a more arctic character than the adjacent sea areas. Brattström (1941) showed that the echinoderm fauna in the 1930s to a significant part consisted of Arctic-Boreal species in the Sound and the southern Kattegat. There are currently examples of cold-water species, common in the Arctic region (Stewart, Pocklington’z & Cunjaki, 1985; Kędra et al., 2010), which are almost only abundant in the Sound and the southern Kattegat, *i.e.,* the bivalves *Musculus niger*, *Nuculana pernula* and *Macoma calcarea*, and the gastropod *Euspira pallida*. *Musculus niger, Euspira pallida* and *Nuculana pernula* are on the national redlist (SLU, 2025) and the two formers on the Helsinki Commission redlist (HELCOM, 2025). Moreover, the temperature conditions in the deep water, below the halocline, could be one of the explanations of recent highly diverse *Modiolus* beds and *Haploops* communities with “type stations” for Petersen’s *Modiolus* and *Haploops* communities (Petersen, 1913; Petersen, 1918; Thorson, 1968; Göransson & Karlsson, 1998; Göransson et al., 2010). Both *Haploops*-species and *Modiolus modiolus* are critical foundation species (Sorte et al., 2017) which create three-dimensional habitats for several associated benthic species and with high importance for fishing. The horse mussel *Modiolus modiolus*, the amphipods *Haploops tubicola* and *Haploops tenuis*, and the ophiuroid *Ophiura robusta* are on the national redlist, and the former three are also on the HELCOM redlist. *Modiolu*s beds are also on the Oslo and Paris Conventions list of threatened and/or declining species and habitats (OSPAR, 2025). Both species of *Haploops* and *M. modiolus* are listed as critically endangered on the Swedish Red List (SLU, 2025). The *Haploops* habitat is potentially under threat and in decline in OSPAR region II (OSPAR Commission, 2021).

Furthermore, in and above the halocline, the Sound holds small populations of the two glacial relicts the amphipod *Pontoporeia femorata* and the priapulid *Halicryptus spinulosus*. This depends, most likely, on the special temperature conditions at the very border of the Baltic Sea where they are common.

Amphipods like *Haploops* spp. and *Pontoporeia femorata* have a high nutrient and fat content and are therefore a valuable food source for many organisms (Baeza-Rojano, Hachero-Cruzado & Guerra-García, 2014).

### Regional observations of changes in the fauna

Several factors structure the benthic macrofauna, both abiotic and biotic. Among abiotic factors temperature and depth are probably the most important (Reiss et al., 2011). The west coast of Sweden (Skagerrak) suffered a reduction in benthic invertebrate biodiversity by 32% between the 1980s and 2010s but the Baltic basins (Bothnian Sea, Baltic Proper, and Bornholm Basin) do not show any significant changes in species richness (Garrison et al., 2022).

Especially since the 1980s, changes in the benthic fauna in the Kattegat and the Sound have been linked to eutrophication (Pearson, Josefson & Rosenberg, 1985; Rosenberg & Loo, 1988; Göransson, 2002). The sharp and sudden changes in salinity and temperature in the region have certainly also been important in this context (Rosenberg, Loo & Möller, 1995). Göransson (2002), revisited Petersen’s stations in the Swedish part of the Sound 1990, and pointed to lower biological variation, which could depend on both eutrophication, hypoxia and xenobiotics. However, fewer finds of northern species such as *Haploops* spp. (none in 1990), *Modiolus modiolus*, *Macoma calcarea* and *Nuculana* spp. were recorded compared to the 1910s. In 2004 a revisit of Petersen’s stations in the Danish part of the Sound showed the total absence of several northern species compared to the 1910s such as *Artacama proboscidea*, *Euspira pallida*, *Mya truncata*, *Nuculana pernula* and *Ophiura robusta* (Göransson, 2004). Follow-up surveys with towed video in 2022 show recent decreases in the Sound of horse mussel beds (Göransson, 2022).

According to Swedish monitoring programs in the Sound the status for four stations (15–23 m depth) varies between moderate and good according to the EU Framework Water Directive (Lundgren, 2023). No trend in status is reported for nine stations in the depth interval 12–14 m for the years 1998–2022 with status between moderate and poor (Bertilsson Vuksan & Eckeskog, 2024).

Concentrations of heavy metals and traditional organic xenobiotics are generally declining in fauna and sediments in the Sound (Bertilsson Vuksan & Eckeskog, 2021).

Several studies have demonstrated impact of bottom trawling on the fauna in the neighboring Kattegat (Pommer, Olesen & Hansen, 2016; Josefson et al., 2018; Sköld et al., 2018; Sköld et al., 2025).

Biotic factors are more difficult to study, but predation is most likely of great importance for the benthic fauna (Moksnes et al., 2008). The ctenophore *Mnemiopsis leidyi* has great impact on the ecosystem by predation on zooplankton (Purcell et al., 2001) and is also a powerful predator on pelagic benthos larvae (Ivanov et al., 2000). The number of reported observations to the Swedish Species Information Centre has increased sharply since 2020 (SLU, 2025). Sköld et al. (2025) showed that predation from fish and Norway lobster is important for structuring the benthic fauna in southern Kattegat. This is especially important for the dominating brittle star *Amphiura filiformis* which appears to benefit from trawling through reduced predation.

## Materials & Methods

### Sampling of benthic fauna

**Figure 1:**
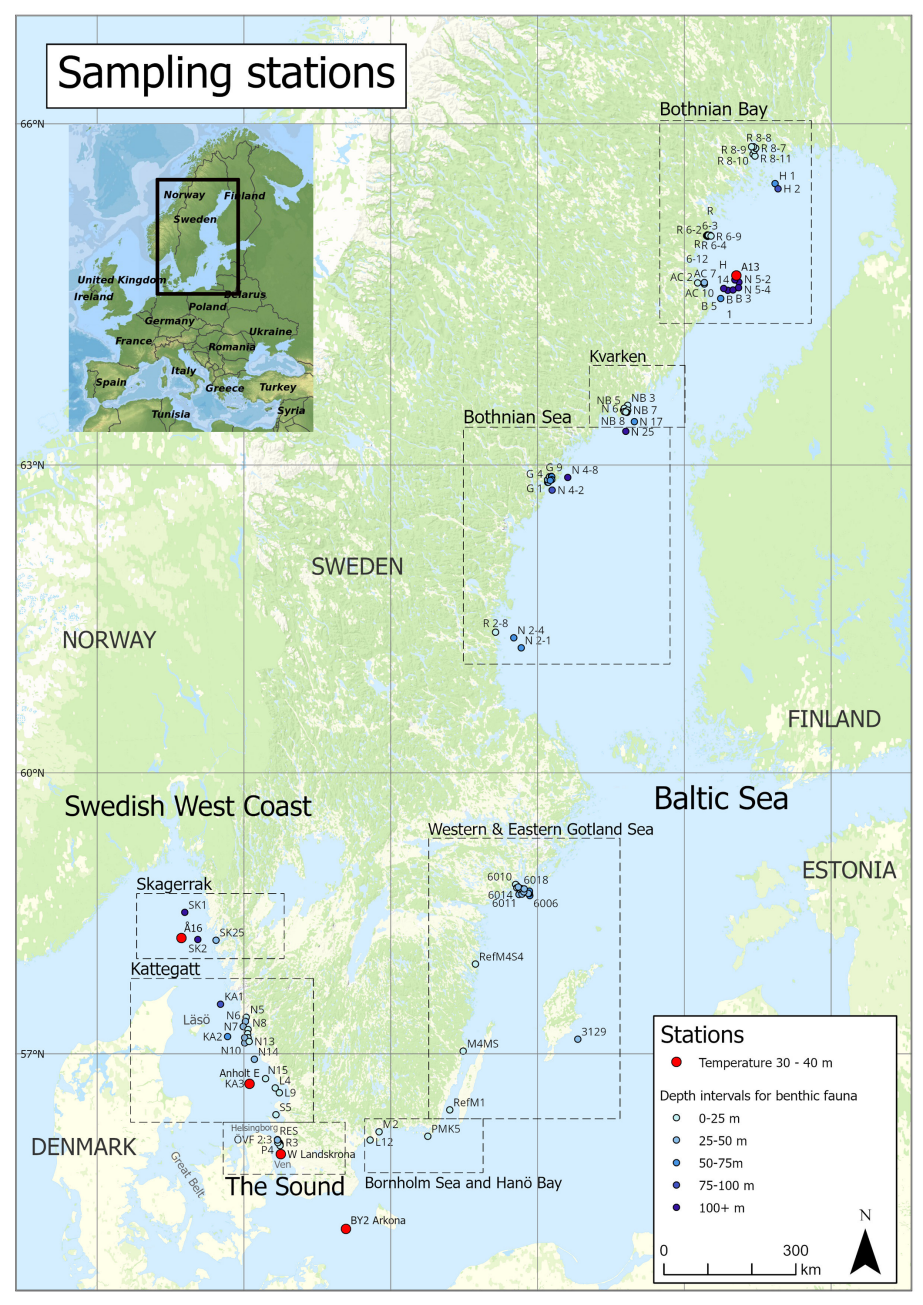
Location of sampling stations along the Swedish coast. Inserted: the studied area in a European perspective. Large map: open data license Creative Commons, CC0 (nytt fönster). Small overview map: copyright: stepmap.

Three very different neighboring areas were chosen for comparison, the Sound, the Swedish west coast (Kattegat and Skagerrak) and the Baltic Sea. Benthic fauna data 1998/2000–2022 was chosen from 12 sampled stations in the Sound (2,551 samples), 20 stations at the Swedish Coast (1,958 samples) and 71 stations in the Baltic Sea (2,229 samples) (Fig. 1). Sampling was performed once a year but stations with nearly continuous time series were chosen, where only four years’ absence was accepted. The total macrofauna dataset includes 6,738 samples and was downloaded from Sharkweb, the Swedish National Database completed with some data from the Helsingborg municipality monitoring program. Sampling in the Sound was 2000–2022 performed with a modified Smith-McIntyre grab with a sampling area of 0.1 m^2^ on depths of about 30 m and with a Haps corer 1998–2022 (Kanneworff & Nicolaisen, 1973) with a sampling area of 0.0125 m^2^ at depths of about 13 m. The difference in sampling strategy reflects the relatively large sized soft bottom marine fauna at about 30 m depth below the halocline, and the relatively small sized brackish water fauna on generally coarser sediment at about 13 m depth. Sampling at the Swedish west coast were performed with a modified Smith-McIntyre grab and in the Baltic Sea with a Van Veen grab, both with a sampling area of 0.1 m^2^. All samples were sieved through a 1.0 mm sieve. The samples were transferred onboard in 4% formaldehyde or 95% ethanol.

Replicate samplings were performed continuously on all stations in the Sound. Five replicates were taken using the Smith-McIntyre grab and ten samples using the Haps corer on the same positions at each station. At the Swedish west coast and in the Baltic the number of samples per station varied between 1 and 5 and decreased in recent years.

Therefore, the most stable sampling was performed in the Sound. This is a good reason to take this as a starting point when choosing species. The Sound is also unique, situated on the border between the west coast and the Baltic Sea. Therefore, the fauna in the Sound is of special interest for studies also from a climatic perspective.

### Temperature and hydrography data

Long term temperature datasets are from five stations; W Landskrona in the Sound, Anholt in the Kattegat, Å16 in the Skagerrak, BY2 in the southern Baltic (Arkona Sea) and A13/F9 in the northern Baltic (Bothnian Sea) and were acquired from the Swedish Meteorological and Hydrological Institute (SMHI, 2024). This was done to provide a rough estimate of the difference and development in the temperature at 30–40 m depth (bottom water in the Sound) between the three areas.

### Processing of data

The choice of species has been based on significant changes in the Sound as sampling was stable and continuous.

Data from each year was pooled to provide a single measure of individuals per sample for the different areas. This was chosen as a method to make an approximate comparison between the continuous replicate sampling in the Sound with various sampling efforts in the other areas. Overall, the data is dominated by unreplicated nonparametric time series. Therefore, the number of individuals has been summed for each species and divided by the number of samples, to give an annual value of the number of individuals per sample in the three areas.

The period 1998/2000–2022 was chosen with reference to data from the Sound and the fact that the data from the other areas is relatively complete. The datasets from the west coast and the Baltic are more incomplete after 2017 when major changes of the Swedish national program were implemented.

All, well defined, species were roughly divided into four major groups according to their main geographical area of distribution (Ekman, 1953). These groups include Arctic-Boreal (“northern”) species, Boreal (“native, or intermediate”) species, Mediterranean-Boreal (“southern”) species and species with a wide area of distribution. The division follows primarily (Brattström, 1941) and Ekman (1953). Here, Arctic-Boreal species are defined as species occurring in the Arctic region as well as the Boreal region (50–90° N). Boreal species are defined as species mainly occurring from northern Scandinavia to the southern North Sea including the English Channel (50–70° N). Mediterranean-Boreal species are species occurring in the Boreal region and into the Mediterranean (30–50° N). Species distributed in all regions were considered widely distributed. In a European Commission perspective the Boreal region in this study covers the Norwegian Sea to the Arctic Circle, the Baltic Sea, the North Sea and the Celtic Sea (http://ec.europa.eu/maritimeaffairs/atlas/maritime_atlas/). The purpose of this rough division is mainly to get an idea of areas of distribution weighed towards north or south relative to the Boreal region. Occasional somewhat isolated occurrences were therefore not considered.

Areas of distribution for most species were given by exact points in the World Register of Marine Species (http://www.marinespecies.org/).

Mode of reproduction was given by Thorson (1946), Kornicker (1988), Hartmann-Schröder (1996), Janssen, Wennberg & Budd (2009) and Capa, Osborn & Bakken (2016).

All trends of species were primarily tested by the nonparametric Mann-Kendall test, but linear regression was also used in long term series graphs to give an idea of the development in relation to a linear trend.

## Results

### The long-term development of benthic species in relation to geographic distribution 2000–2022

Trends 1998/2000–2022 for species in the Sound compared to the Swedish west coast and the Baltic Sea were very similar but fewer comparisons could be made with the Baltic Sea (Table 1). This is because of few species occurring there. Only one species of the selected was recorded for a significant change in the Baltic Sea during the period.

**Table 1:** Nonparametric trends 1998–2022 for species in the Sound compared to the Swedish west coast and the Baltic Sea. Larvae type for different species (non-pelagic *e.g.*, non pelagic lecithotrophic or very short pelagic). The division in Zoogeographical groups see Methods.

Species	The Sound		The West Coast		The Baltic		Zoogeograhical	Type of larvae
	Kendall’s tau	*p*	Kendall’s tau	*p*	Kendall’s tau	*p*	Group	
Decreasing in the Sound								
*Ampharete baltica*	−0.372	0.013	−0.113	NS			Arctic-Boreal	Unknown
*Diastylis rathkei*	−0.541	0.001	−0.012	NS			Arctic-Boreal	Non pelagic
*Dipolydora quadrilobata*	−0.331	0.021					Arctic-Boreal	Non pelagic
*Halicryptus spinulosus*	−0.435	0.007			−0.060	NS	Arctic-Boreal	Non pelagic
*Haploops tenuis*	−0.764	<0.0001	−0.385	0.027			Boreal	Non pelagic
*Haploops tubicola*	−0.616	<0.0001	−0.509	0.001			Arctic-Boreal	Non pelagic
*Maera loveni*	−0.549	0.002	−0.342	0.031			Arctic-Boreal	Non pelagic
*Musculus niger*	−0.371	0.020	−0.335	NS			Arctic-Boreal	Non pelagic
*Nuculana minuta*	−0.704	<0.0001	−0.164	NS			Arctic-Boreal	Non pelagic
*Nuculana pernula*	−0.647	<0.0001	−0.703	<0.0001			Arctic-Boreal	Non pelagic
*Ophiocten affinis*	−0.616	<0.0001	−0.509	0.001			Arctic-Boreal	Pelagic
*Ophiura albida*	−0.724	<0.0001	−0.003	NS			Wide	Pelagic
*Ophiura robusta*	−0.641	<0.0001	−0.268	NS			Arctic-Boreal	Pelagic
*Philomedes brenda*	−0.837	<0.0001	−0.806	<0.0001			Arctic-Boreal	Non pelagic
*Pontoporeia femorata*	−0.505	0.002			0.265	NS	Arctic-Boreal	Non pelagic
*Priapulus caudatus*	−0.468	0.004	−0.293	NS			Arctic-Boreal	Non pelagic
*Sphaerodorum gracilis*	−0.553	<0.0001	−0.393	0.005			Arctic-Boreal	Unknown
*Trochochaeta multisetosa*	−0.396	0.011	0.086	NS			Boreal	Pelagic
Increasing in the Sound								
*Amphiura filiformis*	0.311	0.006	0.448	<0.001			Mediterranean-Boreal	Pelagic
*Cylichna cylindracea*	0.563	<0.0001	0.597	<0.0001			Mediterranean-Boreal	Pelagic
*Diplocirrus glaucus*	0.386	0.012	0.154	NS			Mediterranean-Boreal	Unknown
*Hediste diversicolor*	0.635	<0.0001			−0.471	0.001	Mediterranean-Boreal	Non pelagic
*Notomastus latericeus*	0.449	0.007	0.504	0.001			Wide	Pelagic
*Nucula nitidosa*	0.673	<0.0001	0.636	<0.0001			Mediterranean-Boreal	Pelagic
*Phoronis muelleri*	0.532	0.001	0.439	0.004			Mediterranean-Boreal	Pelagic
*Spiophanes kroyeri*	0.610	<0.0001	0.399	0.008			Wide	Pelagic
*Tritia nitida*	0.040	0.006					Mediterranean-Boreal	Pelagic

The declining species were significantly more than the species found to be increasing and accounted for two-thirds of the changes.

**Figure 2:**
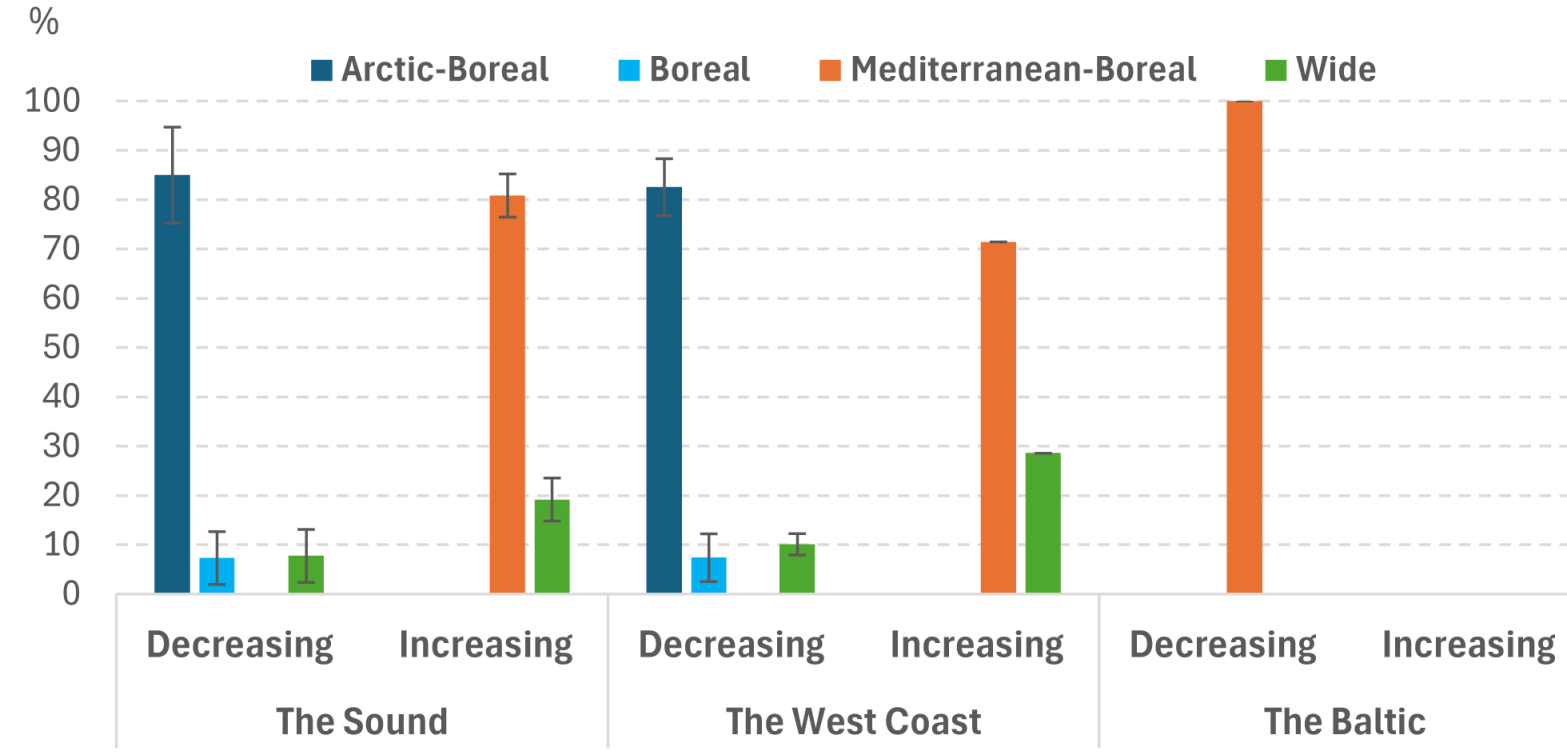
Percentage of species for different area of distribution in different studied sea areas with a significant change in abundance in the Sound 1998–2022. Error bars indicate standard deviation.

**Figure 3:**
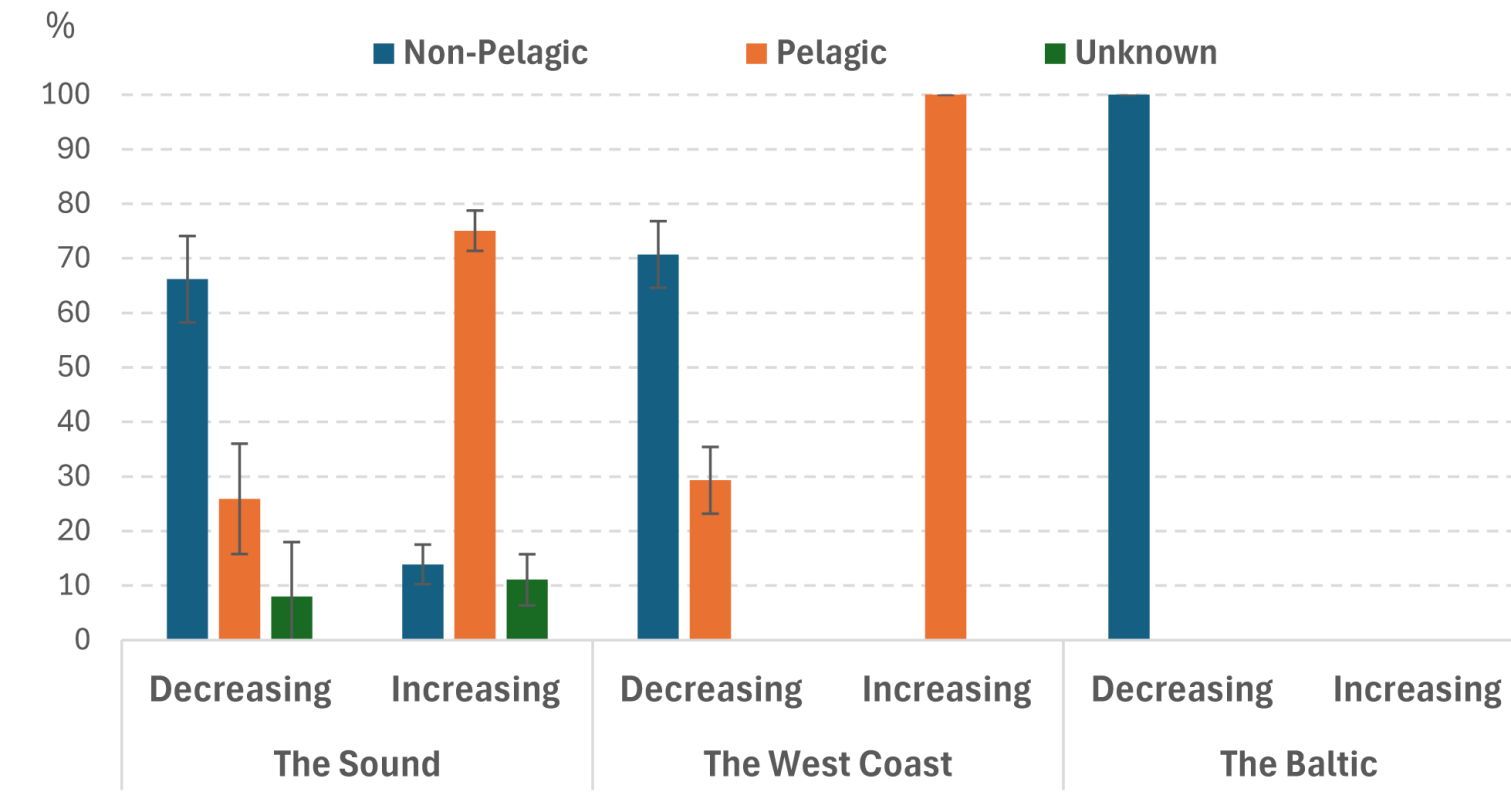
Percentage of species for different type of larvae in different studied sea areas with a significant change in abundance in the Sound 1998–2022. Error bars indicate standard deviation.

Percentage of species in the Sound with a significant change in abundance for different areas of distribution differed very much (Fig. 2). The species that declined were almost exclusively Arctic-Boreal (ANOVA on ranks, *p* < 0.001). Conversely, the species that increased were mainly Mediterranean-Boreal (ANOVA on ranks, *p* < 0.001). In the Baltic Sea, only one of the selected species was noted as increasing, no species was noted as decreasing. A clear predominance of non-pelagic larval stage for decreasing species and an even clearer predominance of pelagic larvae for increasing species was observed (ANOVA on ranks, *p* < 0.001) (Fig. 3). The dominance of non-pelagic larvae for decreasing species was also recorded in all three areas. On the contrary, pelagic larvae dominate the increasing species in the Sound and at the West Coast while no increasing selected species was noted for the Baltic Sea.

The species that declined were almost exclusively northern (Arctic-Boreal). Conversely, the species that increased were mainly southern (Mediterranean-Boreal). In the Baltic Sea, only one of the selected species was noted as increasing, no species was noted as decreasing.

A clear predominance of non-pelagic larval stage for decreasing species and an even clearer predominance of pelagic larvae for increasing species was observed (Fig. 3). The dominance of non-pelagic larvae for decreasing species was also recorded in all three areas. On the contrary, pelagic larvae dominate the increasing species in the Sound and at the West Coast while no increasing selected species was noted for the Baltic Sea.

**Figure 4:**
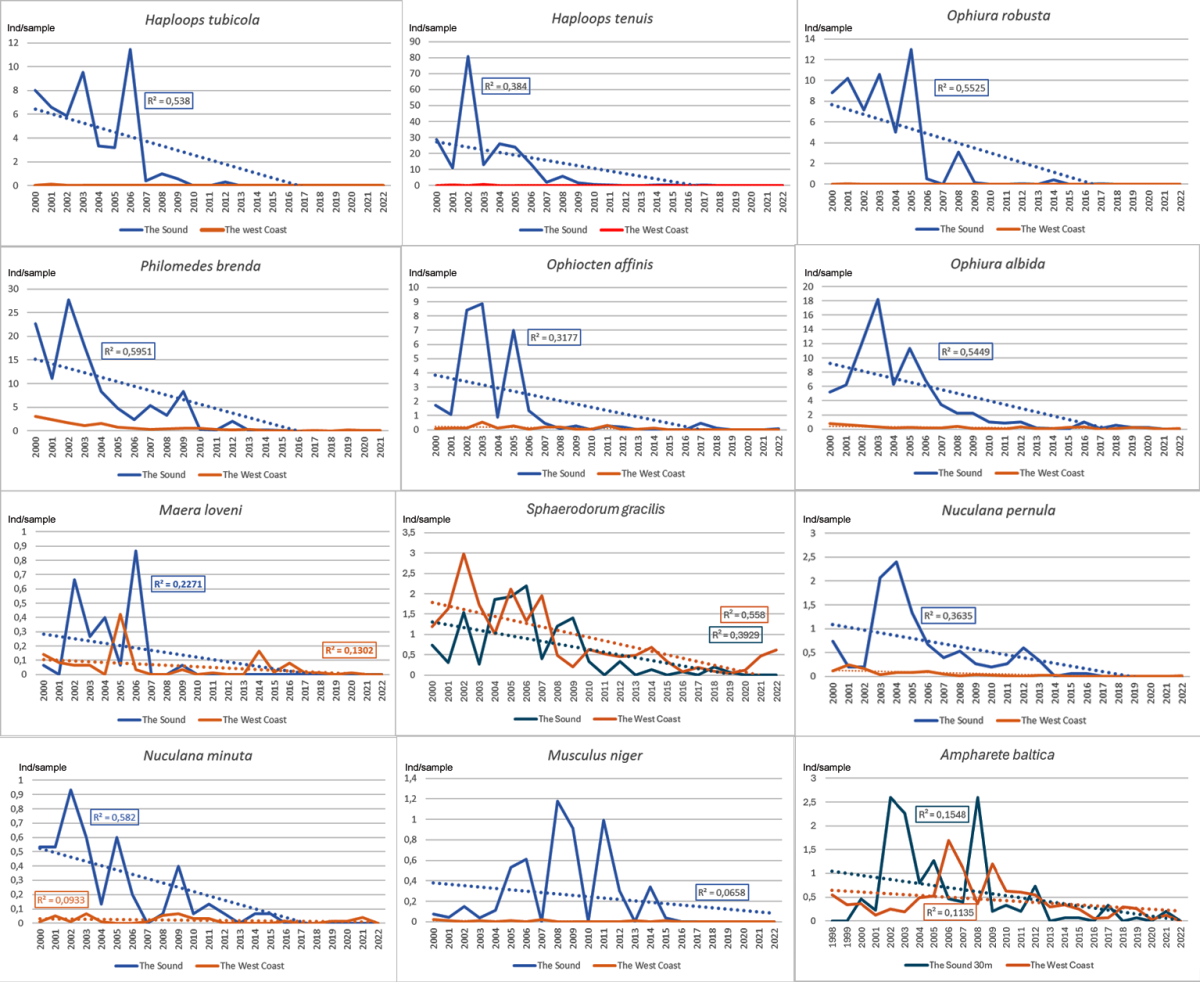
Species with a decreasing development pattern in the Sound at 30 m depth 1998/2000–2022 compared to the Swedish west coast. Number per sample. Trend lines for linear regression.

Species with a decreasing development in the Sound at 30 m depth compared to the west coast show a generally similar pattern in both areas (Fig. 4). Eight of 12 species show relatively high numbers until 2006–10 and are thereafter sharply declining. Four species show a more prolonged decline. Numbers per sample are generally higher in the Sound compared to the west coast.

**Figure 5:**
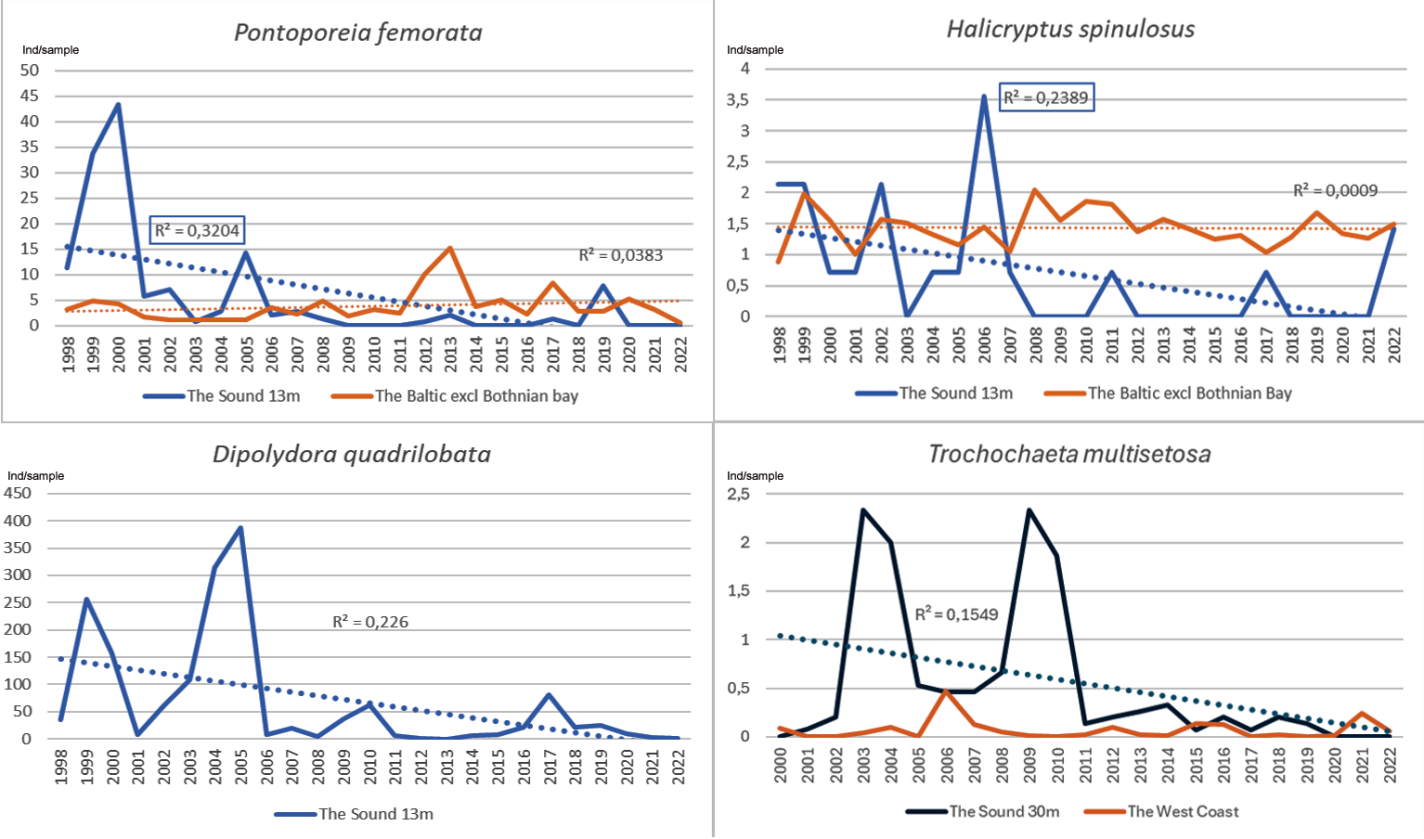
Species with a decreasing development pattern in the Sound at 13 m depth 1998/2000–2022. *P. femorata* and *H. spinulosus* compared to the Baltic. *T. multisetosa* compared to the Swedish west coast. Number per sample. Trend lines for linear regression.

Species with a decreasing development in the Sound at 13 m depth have remained relatively steady in the Baltic (Fig. 5).

**Figure 6:**
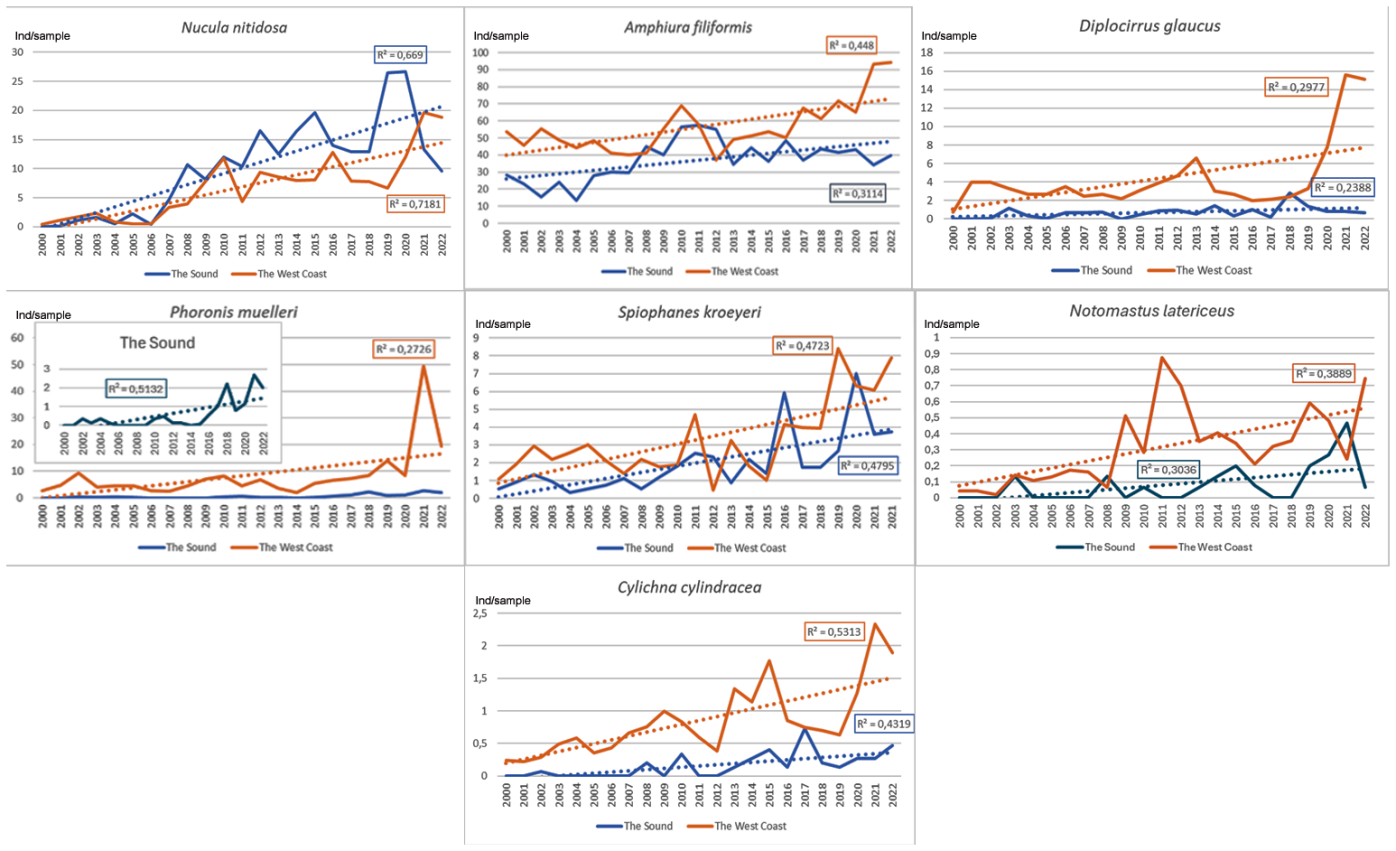
Species with an increasing development pattern in the Sound at 30 m depth 2000–2022 compared to the Swedish west Coast. Number per sample. Trend lines for linear regression.

Species with an increasing occurrence in the Sound at 30 m depth compared to the west coast showed different patterns (Fig. 6). Five of seven species showed a more or less linear increase throughout the period in both areas. Two species showed an increase in recent years in both areas. The number per sample was generally higher on the west coast. Most species at about 30 m depth that increased during the period 2000–2022 roughly show the same successive development pattern. However, the polychaete *Diplocirrus glaucus* and the phoronid *Phoronis muelleri* deviated*.* They increased in abundance, mainly during the last years of this study. The increase of the regionally dominant brittle star below the halocline, *Amphiura filiformis*, was reduced since about 2012 in the Sound but continued sharply along the west coast. The species with an increase in abundance was more pronounced on the west coast than in the Sound.

**Figure 7:**
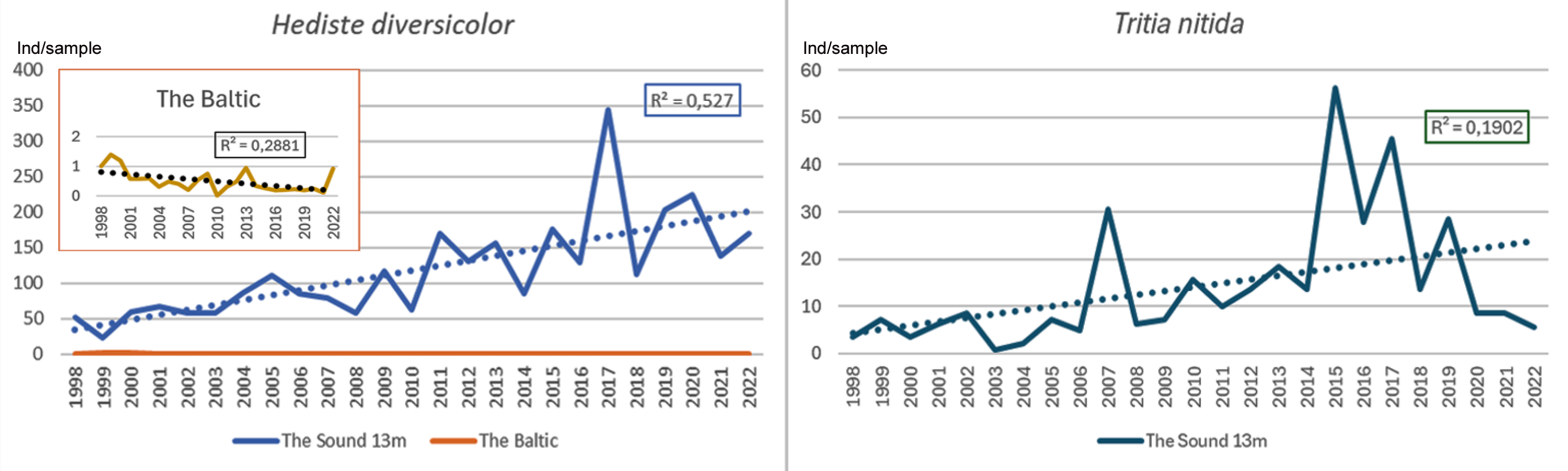
Species with an increasing development pattern in the Sound at 13 m depth 1998–2022 compared to the Baltic (*Hediste diversicolor*) and *Tritia nitida* (data only from the Sound). Number per sample. Trend lines for linear regression.

Species with an increasing development in the Sound at 13 m depth compared to the Baltic showed about the same linear increase in the Sound (Fig. 7). However, the polychaete *Hediste diversicolor* showed a significant decreasing trend in the Baltic. An increasing trend was recorded for the gastropod *Tritia nitida* in the Sound. The number per sample of *Hediste diversicolor* was much higher in the Sound than in the Baltic.

The priapulid *Priapulus caudatus* and the cumacean *Diastylis rathkei* both decreased in abundance in the Sound at 13 m and 30 m depth. However, on the west coast no significant decrease was noted. For both species the number per sample was highest in the Sound at 13 m depth.

**Figure 8:**
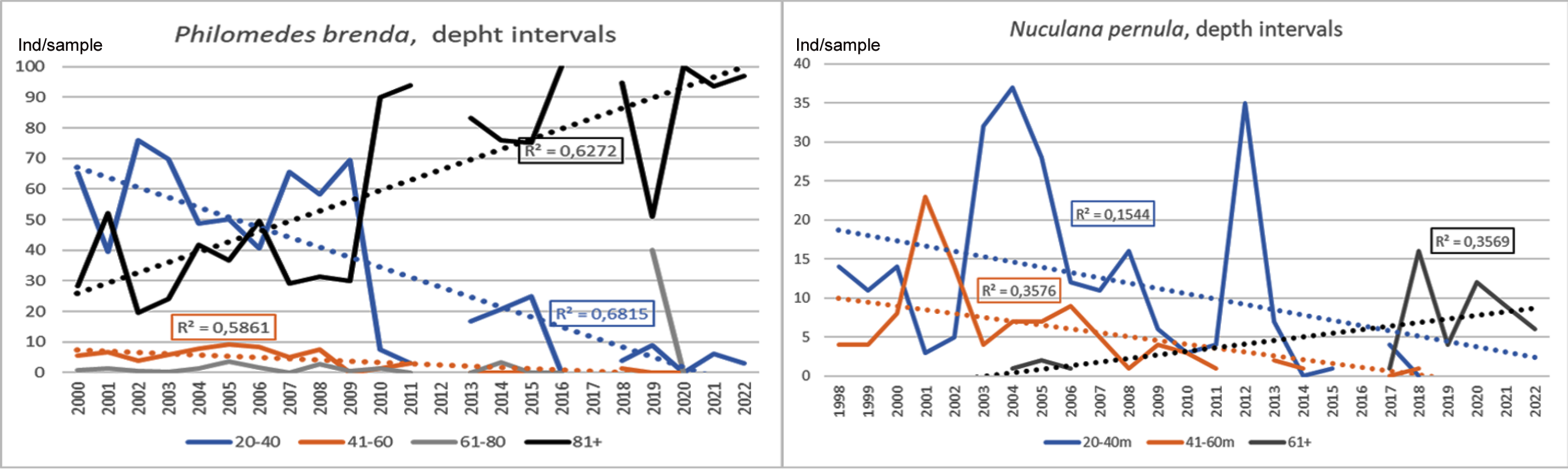
Development for *Philomedes brenda* and *Nuculana pernula* at different depth intervals in the Sound and the Swedish west Coast together 2000–2022. Number per sample. Trend lines for linear regression. Total data for the Swedish coast 2000–2022.

In the Sound and on the west coast the ostracode *Philomedes brenda* and the bivalve *Nuculana pernula* both showed a similar development in abundance between 2000 and 2022 at different depths (Fig. 8). Both species showed declining trends at lower depth intervals and increasing trends at deeper intervals. Both species seemed to decrease at 20–40 m and 41–60 m and increase at more than 81 m respectively to 61 m. Data between years in this special case is less comparable than otherwise. As data is scarce for those species, it is impossible to get relevant time series for the selected stations, total data for the Swedish coast has been used. This indicates a certain bias in the comparison.

### Bottom temperature

Temperature data from five different stations along the Swedish coast show statistically significant increases at all but one station (Table 2). The highest increase was recorded at 15 m depth, near the halocline, in the Sound. Increases in the Sound 1997–2022 at the 15 to 40 m level were on average 0.94 degrees.

**Table 2:** Changes in bottom temperature for five stations along the Swedish coast 1997–2022. Linear regression of annual means. Data from Swedish Meteorological and Hydrological Institute.

AREA, Station	r^2^	*p*	Change per year °C	Total change °C
THE WEST COAST Å16, 30 m	0.183	0.029	+ 0.026	+ 0.67
THE WEST COAST Anholt, 30 m	0.207	0.042	+ 0.031	+ 0.81
THE SOUND W Landskr. 30 m	0.235	0.012	+ 0.026	+ 0.67
THE SOUND W Landskr. 15 m	0.181	0.030	+ 0.053	+ 1.38
THE BALTIC BY32 30 m Mean 30 m	0.093	NS	+ 0.048	+ 1.23
THE BALTIC F9/A13 40 m	0.312	0.003	+0.034	..+ 0.88

**Figure 9:**
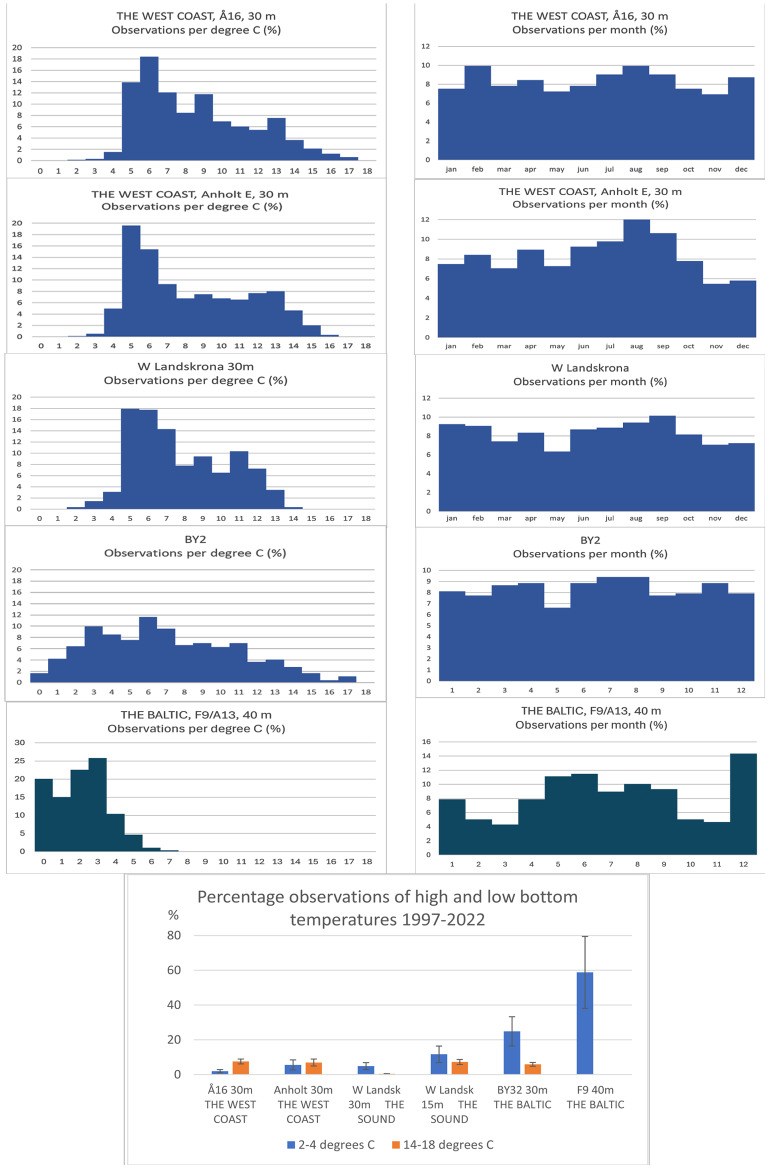
Temperature conditions 1997–2022 for five stations along the Swedish coast. Stations arranged from north on the west coast to the south in the Sound and further into the Baltic Sea. Percentage observations per degree C and percentage observations per month. At the bottom: Percentage observations of high and low bottom temperatures.

Temperature conditions differed for the five stations (Fig. 9). Percentage observations per degree show a slightly increased proportion of observations with low temperatures (2–4 °C) from the west coast towards the Sound and then a strong increase of observations of low temperatures into the Baltic Sea (linear regression, *r*^2^ = 0.604, *p* < 0.001). Station F9 in the Gulf of Bothnia is the only station that differs significantly from the others (ANOVA, *p* < 0.041). When it comes to high temperatures (14–18 °C), F9 and the Sound at 30 m depth occupy a special position with no or very few observations of high temperatures. The narrowest temperature range was recorded for the Sound and the northern Baltic Sea.

Observations per month are fairly evenly distributed over the various months for most stations. Station F9, in the northernmost Baltic, deviates with relatively few observations during spring and autumn and relatively many observations in December. The latter implies a certain bias for results per degree for this station. Percentage observations of high and low bottom temperatures show an increasing trend towards lower temperatures towards the Baltic Sea. High temperatures are most unusual in the Sound and furthest north in the Baltic Sea.

## Discussion

### Comparability of benthic fauna samples

The data series differ in quality between the areas. This is mainly caused by the change in sampling strategy with changed positions and replication in connection with the introduction of status classifications according to the European Framework Directive. Valuable time series along the Swedish coast have been broken and make relevant comparisons between different areas in time difficult or even impossible. This is a serious problem as the contribution for long-term ecological and environmental studies of how species and ecosystems respond to a changing global climate becomes more urgent (Hughes et al., 2017; ICES, 2024). Regarding the effects of bottom trawling on benthic fauna, it has been shown that several indices, used to classify status according to the European Water Framework Directive, can give a completely misleading picture of the conditions (McLaverty et al., 2023). The sampling configuration and the indicator used may to a large extent determine the results of the Ecological Status (Karakassis et al., 2013). Furthermore, and most important, the increasingly frequent use of different indices also sometimes means that changes in species composition are overlooked. The indices rarely consider what is normal for a certain area. This is a significant problem because of the great variation between different geographical areas. The indices only provide a single artificial value of biological diversity and cannot replace the studies of individual species. This often means that relevant information is not used and therefore risks misinterpretation of results.

In this study there are major differences in the continuity of the data series and replication of samples between the different areas. Complete data series with replicates for the period 1998–2022 are only available from 12 stations in the Sound and 13 stations in southern Kattegat. No complete series exist from the northern part of the west coast where replication almost ceased in 2013. From the Baltic Sea there are 39 complete data series of which only four with three replicates. This means that the relevant data material has a strong bias towards the Sound and southern Kattegat. The large difference in the size of the areas, the small Sound compared to the much larger west coast and the huge Baltic Sea, means that local differences more or less are overshadowed in the larger areas. The use of different samplers in the Sound and in the Baltic is also significant. The use of Haps-corer generally results in higher abundances than for Van Veen grab (Jensen, 1981). The variation in abundance must be seen against this background. However, a large data set was chosen, and the sample area consists of different soft bottom associations. This should mean relatively good comparisons regarding long-term distribution and development of the species. Furthermore, several redlisted species (decreasing and threatened) were noted. A high proportion of these species have a northern area of distribution (Göransson, 2017a) which makes them especially interesting to study from a climate perspective.

### Long term development pattern of abundance

The individual species that changed significantly over the 25-year period showed no clear cyclical pattern. However, the 7–8-year cycles usually given for the NAO mainly apply to the variation in total abundance (Tunberg & Nelson, 1998).

**Figure 10:**
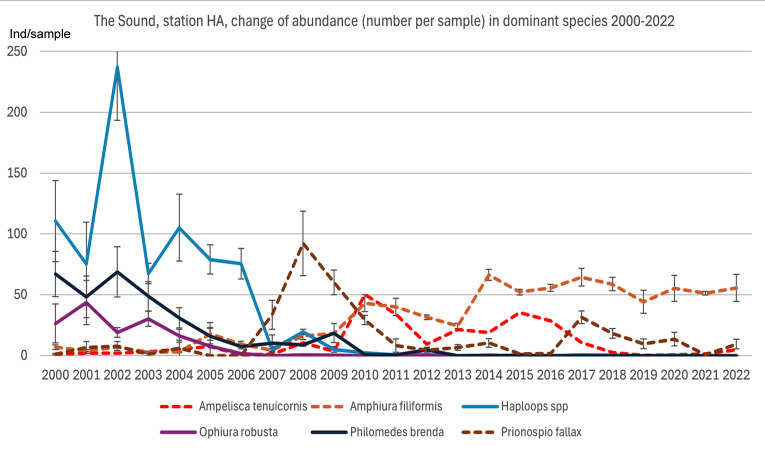
The transition from a *Haploops* community to an *Amphiura* community. Change of dominant species 2000–2022, station HA in the Sound. Mean number per sample and standard error each year. Data from the Helsingborg monitoring program.

A majority of species with a decreasing abundance in the Sound at 30 m depth and the west coast shows a generally similar pattern with relatively high numbers until 2006–2010. However, thereafter a sharp decline was recorded. The recovery after this decline appears to be absent or very modest in the following years. This is an indication of common impact factors in the Sound and on the west coast. However, the change was highest in the Sound, which may be due to a greater impact response. Eight species in both areas seem to have been acutely impacted. Half of these can be considered to belong to the *Haploops* community (*Haploops* spp., *Ophiura robusta*, *Ophiura albida*, *Philomedes brenda* and *Nuculana pernula*). The reduction of *Haploops* at the only continuously monitored *Haploops* station in the Sound has meant a long-term change in the dominance conditions. This is an indication of a transition from a *Haploops* community to an *Amphiura* community (Fig. 10). This process may be a continuation of what happened at the *Haploops*-community in the late 1900s in the southern Kattegat, where the brittle star *Amphiura filiformis* now dominates. After the sharp decline in 2007 in the Sound of *Haploops* and the associated northern species, several southern species increased here. Primarily an almost opportunistic peak of the polychaete *Prionospio fallax* and then an increase of the amphipod *Ampelisca tenuicornis*. Finally, *A. filiformis* increased and then stabilized at a relatively high level and became the highly dominant species. The large variation of *Haploops* indicates that it may be at risk of extinction at low population levels. The interannual variation is strange considering that it has non-pelagic larval development, which usually means a relatively stable occurrence (Thorson, 1950a). However, in the Sound this may be caused by the fact that it lives close to its distributional boundary, therefore exposed to random events affecting population size (Grayson & Johnson, 2017).

On the contrary, *A. filiformis* has a much more stable occurrence in the region (Muus, 1981) and with its large quantities of pelagic larvae it can gradually expand on bottoms that were previously dominated by *Haploops*. Rigolet, Dubois & Thiebaut (2014) consider that another *Haploops* species, *H. nirae,* controls diversity and abundance of associated species in Brittany. However, the most common associated species in the Sound started to decline nearly 4 years before the dramatic reduction of *Haploops* spp. in 2007.

A more prolonged decline was recorded for the bivalves *Nuculana pernula, Nuculana minuta* and *Musculus niger* as well as for the polychaete *Ampharete baltica*. *N. pernula* has a relatively low metabolism which, for instance, makes it less sensitive to oxygen deficiency (Jones, Kelly & Mincks, 2021).

The reduction in the Sound of the two sparsely occurring typical glacial relics, *P. femorata* and *H. spinulosus*, that are common and relatively stable in the Baltic Sea, is an indication of less favorable conditions for these in the Sound, at their outermost distributional border.

The northern polychaete *Dipolydora quadrilobata*, which mainly was reported from the Sound, showed mass occurrences next to load of nutrients in the early 2000s. The decrease in load could be a sign of a lower recent trophic level.

In the Sound the polychaete *Hediste diversicolor* and the gastropod *Tritia nitida* increased in abundance in a similar way during the entire study period 1998–2022. The increase was stable for *H. diversicolor*. It has a bottom-living larval stage, while *T. nitida*, has a pelagic larva. The decreasing trend for *H. diversicolor* in the Baltic Sea is hard to explain for this very resilient species (Silva et al., 2020; Pham et al., 2024). Different studies (Bartels-Hardege & Zeeck, 1990; Fernandes et al., 2023) showed that temperature is important for spawning. Field observations indicate low winter temperature as beneficial (Beukema, 1990) possibly by reduced predation (Armonies, Herre & Sturm, 2001). The opposite trends in the Sound and the Baltic Sea may be due to differences in temperature regimes between the areas, but most likely because the species has its distribution limit in the Baltic Sea.

Two species with a decreasing development at 30 m depth in the Sound are the priapulid *Priapulus caudatus* and the cumacean *Diastylis rathkei.* However, no statistical change was recorded at 13m depth and on the west coast. This indicates that the changes primarily occurred in the bottom water in the Sound. Tott (2017) predicts submergence in connection with global warming for *P. caudatus*, but this was not observed in the area. *D. rathkei* is mainly controlled by low winter temperatures (Rachor et al., 1982). Persson (1981) reports winter temperatures (1–2 °C) as negative and high winter temperatures (3–4) °C as positive for the southern Baltic Sea which is colder than the west coast and the Sound.

The similar temporal trends for species in the Sound and the west coast were most likely due to the relatively similar fauna here, in contrast to the Baltic Sea, which harbors fewer species. The lower temperature regime in the Baltic Sea also means that relatively more cold-water species occur there. A similar increase in temperature probably therefore has lesser impact on cold water species in the Baltic Sea than in the Sound and along the west coast.

The fact that the majority of the declining species in the Sound have a benthic larval stage and most of the increasing species have a pelagic larval stage is highly significant. This suggests that reductions are due to factors internal to the Sound itself.

Numbers per sample were generally higher in the Sound compared to the west coast for northern species but these species have decreased markedly in the Sound. On the contrary, the abundance of southern species was generally lower in the Sound but has increased. This confirms the general notion that the fauna nowadays has a “northern character”, but also that this character has diminished.

### Historical perspective on change in species composition

Within a relatively large area in the southeastern Kattegat, roughly about 1,000 km^2^, scattered occurrences of the *Haploops*-community were recorded in the beginning of the 1900s (Petersen, 1913). Jägerskiöld (1971) sampled 440 stations on the west coast with dredge 1921–1938 and reports 20% of the stations with *Haploops spp*. Thorson (1968) verified Petersen’s area in the 1960s and mean abundance of *Haploops* spp. in this area was about 2,000 individuals per m^2^, a relatively high number for those Arctic-Boreal species compared to a maximum of 103 ind/m^2^ present time at around 70° N (Conlan, Hendrycks & Aitken, 2019). Petersen’s “type-station”, 22, on the northern edge of the Sound, was revisited for the first time in 1990, but no *Haploops* were found (Göransson, 2002). Additionally in 1985–2018 when a few other *Haploops*-stations in the southern Kattegat were revisited only a few individuals were encountered (Pearson, Josefson & Rosenberg, 1985; Göransson, 1999; Sköld et al., 2018). A sharp decline of *Haploops tubicola* was also recorded in the Skagerrak between 1937 and 1990 (Miskov-Nodland, Buhl-Mortensen & Høisæter, 1999) and in the Danish Great Belt 2001 (J Hansen, pers. comm., 2014). In the Kattegat *Haploops* spp. were only recorded at a single station at 60 m depth in 2016 despite extensive sampling both with bottom grab and towed video camera (Dinesen et al., 2020). The latter is strong indications of a long-term decline in the *Haploops*-community in a large area.

Observations of *Haploops* in the Sound goes at least all the way back to *Liljeborg*, who named the species *tubicola* (1856); Meinert (1877); Lönnberg (1903); Björck (1915); Stephensen (1916); Eliason (1920); Dahl (1946); Kanneworff, who named the species *tenuis* (1966); Persson & Olafsson (1980) and Göransson (1995, unpublished data 1988–2024). Between 104 and 10.100 ind/m^2^ were recorded. From this perspective, the dramatic reduction in 2007, at the only regularly monitored station north of the island Ven, appears historic (Göransson et al., 2010). At a follow up study at 49 stations around Ven in 2010 only single individuals of *Haploops* were found (Jönsson, 2011; Nordh, 2011; Tytor, 2011). Since 2014 very few individuals of *Haploops* have been recorded in the Sound and on the Swedish west coast.

**Figure 11:**
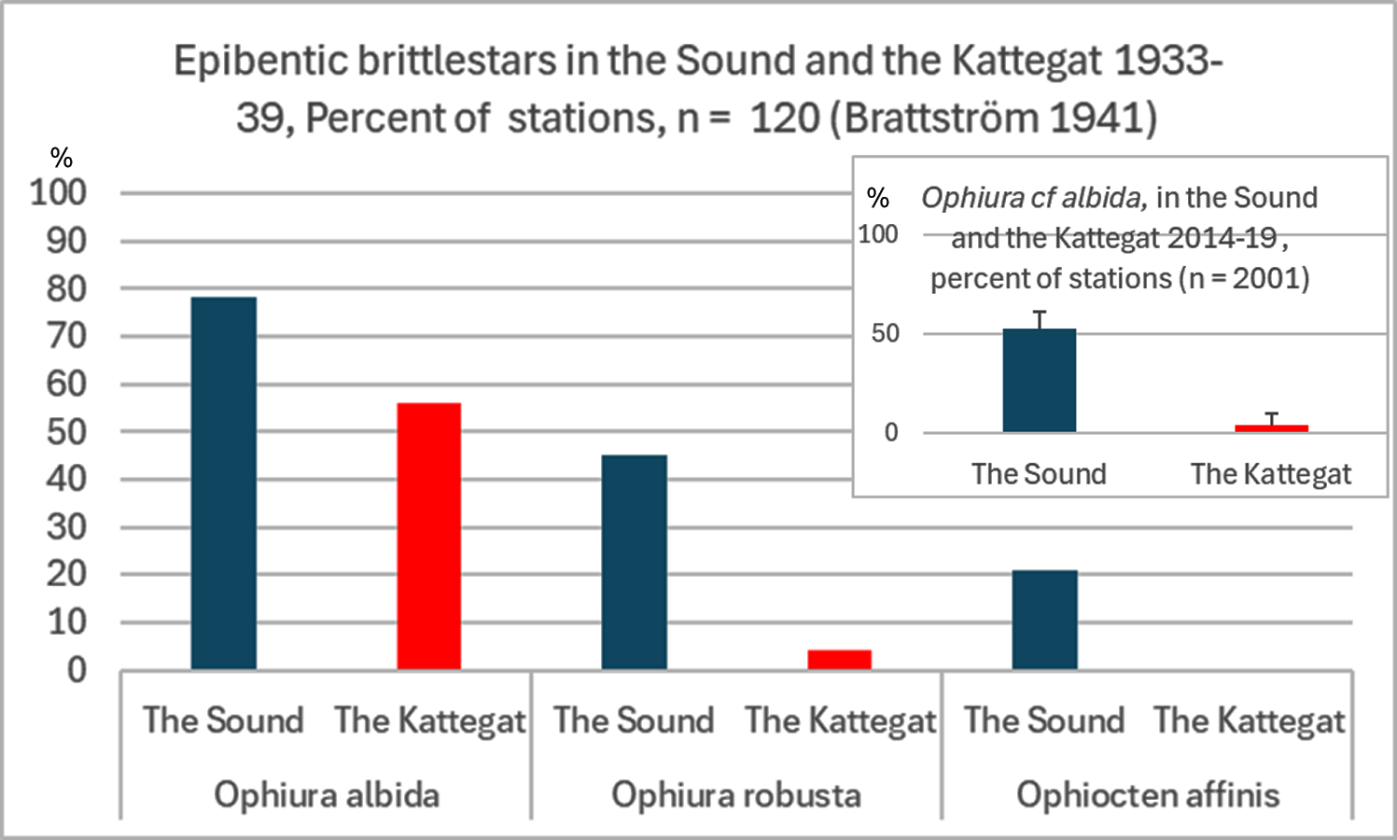
Epibenthic brittlestars on soft bottom in the Sound and the Kattegat 1933–39 and 2014–19. Compiled data from dredge-samplings 1933-39 (Brattström, 1941, *n* = 120). Inserted compiled data for *Ophiura* cf *albida* from towed video recordings 2014–19 (*n* = 2,001). Mean and standard deviation.

In addition to the two *Haploops* species, very strong reductions in the 2000s were also observed in the soft-bottom epibenthic brittlestars *Ophiura robusta*, *Ophiura albida* and *Ophiocten affinis.* Jägerskiöld (1971) reports 52% of the stations with *Ophiura albida*, 22% of the stations with *Ophiocten affinis* and 10% of all sampled stations with *Ophiura robusta* for the period 1921–1938. According to Brattström’s dredge samplings 1933–39 (Brattström, 1941), the three common epibenthic ophiuroids in the area, were then significantly more common in the Sound than in the Kattegat (Fig. 11). Even if Brattström’s data is not directly comparable with today’s surveys, major differences appear with the conditions in 2014–2019, when a large number of stations were monitored by towed video (Emanuelsson & Göransson, 2015; Emanuelsson & Göransson, 2016; Göransson, 2017b; Göransson, 2018a; Göransson, 2018b; Göransson, 2019; Göransson, Emanuelsson & Lundqvist, 2014) (*n* = 2,001). These samples are directly comparable because they are based on the same area (25 m^2^).

The historical development in the Sound of the high Arctic bivalve *Nuculana pernula* is similar to several other Arctic-Boreal species. Lönnberg (1899) lists it as common or very numerous. Thulin (1921) therefore believes that it creates its own benthic fauna community, “the Leda pernula community”, in the Sound with abundances up to 74 individuals per sample. In 1979 only one individual per sample was recorded on a few stations in the northern part of the Sound (Persson & Olafsson, 1980). A comparison between 1990 and Petersen’s stations from 1910–12 (Petersen, 1913) shows a decline in four of five stations (Göransson, 2002). The result from recent decades is an indication of a more pronounced decline (Fig. 4). Since 2010 a maximum of ten individuals per year was observed on the whole Swedish coast (SLU, 2025).

The abundance of *Philomedes brenda* also seems to have decreased even longer term. This small, millimeter sized, ostracode can be overlooked in the samples. However, it has almost been a type species in the Sound for the *Haploops* community. In 1979 an average of 73 individuals per sample was recorded on five *Haploops* bottoms in the vicinity of station ÖVF2:3 (Persson & Olafsson, 1980). From 1993–1998 an average of 126 individuals per sample was recorded on a station north of the island of Ven (P Göransson, 1993, 1994, 1995, 1996, 1997, 1998, unpublished data). This relatively inconspicuous species was thus common until recently but has decreased dramatically in the new millennium (Fig. 4).

In summary, the recent decline of many northern species in the Sound has a history that is documented far back.

### Possible explanations for change in abundance and species composition

Bottom trawling, hypoxia, eutrophication and climate change were listed as some possible explanations when the first signs of the reduction of *Haploops* were discovered (Göransson, 1999). The dramatic reduction of *Haploops* in the Sound 2007 was followed by an opportunistic peak of a species not commonly associated. This is a pattern typical of a severe disturbance, for instance oxygen deficiency (Pearson & Rosenberg, 1978). This occurred after the lowest measured value, 0.91 ml/l, for oxygen concentration in the bottom water for the period 1997–2022 was recorded in a month-long period, when the levels were well below 2.0 ml/l. This is a level where negative effects on the benthic fauna can be expected (Diaz & Rosenberg, 1995) and some amphipods seem especially sensitive (Wu & Or, 2005). Also, oxygen concentrations < 1 ml/l were measured several times in the bottom water in the main distribution area for *Haploops* in southern Kattegat 1987 and 1988 in connection with mass mortality of benthic fauna (Diaz & Rosenberg, 1995). Hypoxia can alter the larval settlement pattern and result in a shift in the benthic invertebrate community (Cheung et al., 2014). *Haploops* spp. are probably sensitive to oxygen deficiency (E Kanneworff, pers. comm., 2024), but the reproduction may also be negatively impacted by increasing temperatures as they can be considered cold stenotherm (Enequist, 1949). In 2007 when low oxygen concentrations were recorded in the Sound, minimum temperature was very high (5.72–6.89 °C in Jan–Feb). There is also a strong synergistic effect between temperature and hypoxia because benthic animals are less tolerant to hypoxia at increased temperatures (Dries & Theede, 1974; Eriksson-Wiklund & Sundelin, 2001; Altieri & Gedan, 2014; Schulte, 2015; Earhart et al., 2022).

Trawling may impact the fauna in Kattegat but also impact the recruitment of species with pelagic larvae in the Sound where trawling is banned (Hansen & Andersen, 2024). The Kattegat has been trawled for at least 80 years (Pommer, Olesen & Hansen, 2016), and the intensity is among the highest in Europe (Eigaard et al., 2017). Therefore, it is of interest to compare the conditions of the benthic communities in the beginning of the trawl period in the Kattegat with the Sound, where trawling has been banned since 1932. The soft-bottom epibenthic brittlestars, with northern and wide distribution, which decreased clearly in recent times have a pelagic larval stage, and they live on top of the sediment surface. Therefore, they are exposed to both predation and bottom trawling. The southern *Amphiura filiformis*, on the other hand, lives buried in the sediment, and this species seem to have increased in abundance. The major difference between the rich occurrences in Sound with its trawl ban and the low incidence in the heavily trawled Kattegat can be interpreted as bottom trawling reduces the abundance of epibenthic brittlestars, but there was a difference between areas before heavy trawling started. Although the data is not directly comparable over time, the occurrences appear to have decreased sharply in both areas and relatively most in the Kattegat. In accordance, these brittlestars were almost absent in grab samples from the Kattegat 2009–2014 (Sköld et al., 2018). However, Rumohr & Kujawski (2000) reported a marked increase in *Ophiura albida* in a trawled area of the southern North Sea in 1986 compared to pristine conditions in 1902–1912. Kaiser et al. (2000) compared fauna in areas that have been exposed to either high or low levels of bottom-fishing disturbance. The results indicated that chronic fishing caused a shift from communities dominated by relatively sessile, emergent, high biomass species to smaller-bodied species such as *Ophiura albida*. The completely different results from these studies could be due to differences between the areas in environmental factors and the impact of trawling or predation. Although the environment in the Sound probably favors these epibenthic brittlestars, the major difference between the areas is striking. The great variation in substrate but above all, the lower temperature in the bottom water, may be the basic explanation for the relatively high individual densities in the Sound. Neumann, Ehrich & Kröncke (2008) and Kröncke et al. (2019) reports very high abundance 1998–2000 of *Ophiura albida* in the North Sea in connection with the cold winter 1995/1996 followed by a continuous decrease from 2000 due to an increase in temperature. Also, the high abundance of epibenthic brittlestars in the relatively cold winters in the Sound 2000–2005 stands in sharp contrast to the low abundance in the generally warm winters 2010–2022. Thus, bottom water temperature may be an important factor in explaining the variation in abundance of the epibenthic soft-bottom brittle stars in the Sound and the Kattegat.

### Trawling, predation, nitrogen, temperature and *Haploops*

Trawling impacts several species in the Kattegat (Pommer, Olesen & Hansen, 2016; Josefson et al., 2018; Sköld et al., 2018; Dinesen et al., 2020). Above all, large species seem to be negatively affected (Pommer, Olesen & Hansen, 2016; Josefson et al., 2018), but also many tube-building species. It is therefore likely to assume that the decline of *Haploops* spp, at least partially, was caused by this serious disturbance (Dinesen et al., 2020). However, the radical reduction in the Sound with its long-term trawl ban appears primarily to be due to other factors. Trawl fishery has also declined in recent decades in the Kattegat (Pommer, Olesen & Hansen, 2016). Serpetti et al. (2013) consider that trawling reduces predation on *Haploops setosa* and ostracodes in the NE Atlantic and Smith, Ragnarsson & Collie (2025) reports high spatial overlap between bottom fishing effort and fish predation of benthos in US Continental shelf. Predation from fish on benthic fauna has been shown to be significant in the Kattegat and to change species composition (Sköld et al., 2025). Predation from fish should be greater in the Sound with its long-term trawl ban than in the surroundings and can be a complementary explanation for changes in the benthic fauna. However, declining populations of the most important predators, cod *Gadus morhua* and dab *Limanda limanda* (Petersen, 1918; Nordenberg, 1963; Dauvin, 2024), were recorded in connection with the drastic decline in *Haploops* populations in 2007 (Göransson et al., 2010). The cod population in the Sound shows no trend in size structure between 1991 and 2016 (Svedäng & Hornborg, 2017). Moreover, Hall et al. (1990) suggest that neither predation nor disturbance by dabs is likely to be important for the prey community.

Another species of *Haploops*, with distribution from Morocco to South Brittany, *H. nirae*, has extended its habitat over thousands of hectares in shallow waters of South Brittany bays, Bay of Biscay, Atlantic, over the last decades (Rigolet et al., 2012). It is still unknown what has caused the recent expansion, but local eutrophication and offshore bottom-trawling activities could potentially promote further expansion in South Brittany with abundances from 6.800 to 25.500 ind/m^2^ (Rigolet, Dubois & Thiebaut, 2014). Therefore, the increasingly lower levels of nitrogen (Lønborg & Markager, 2021), that mainly control the primary production (Graneli, 1984; Richardson & Christoffersen, 1991), may have created relative food shortages in the Sound. A large-scale regional decline in primary production has earlier been presented by Henriksen (2009) which could depend on both decreased nitrogen load and increased temperature. Lønborg & Markager (2021) considered this decrease to start already in the early 2000s. *Haploops* seems to benefit from a high trophic level (Rigolet et al., 2012). Therefore, this is a possible contributing explanation for the current decline of *Haploops* in the Sound. On the other hand, the intestines were well-filled in most of the *Haploops* examined in this study 2011–13. Also, an increase of several other species does not indicate that a general decline in phytoplankton production is the most important explanation for the changes over the past 25 years.

### Reproduction of *Haploops*

According to Kanneworff (1966) the hatching time for both *Haploops tubicola* and *Haploops tenuis* in the Sound was January–February. This time period is an adaptation to “match” the spring bloom, in order to maximize food availability for the young (E Kanneworff, pers. comm., 2024). Well-developed marsupial eggs were observed from October to January in the Sound in the 1960s. However, in the Gullmarsfjord (northern west coast) they were found in the 1930s already in August-December (Enequist, 1949). The temperature at 25 m in the Sound in 1960 began with a low dip in January at 2–5 °C and increased to 12 °C in December just before hatching. In the Gullmarsfjord the temperature at 60–70 m was 4.3 °C in April and with a maximum of 11 °C already in July–August. Mattson (1990) recorded about the same temperatures 1979 and 1980 in connection with many records of *Haploops* in cod stomachs. The deeper occurrence in Skagerrak compared to the Sound is probably due to the relatively higher bottom temperature here. Enequist (1949) considered *Haploops tubicola* to be a cold-stenothermal species, dependent on a low temperature during the maturation of gonads. Enequist also stated that 100–200 m was a general depth distribution for the Skagerrak compared to about 30 m in the Sound.

**Figure 12:**
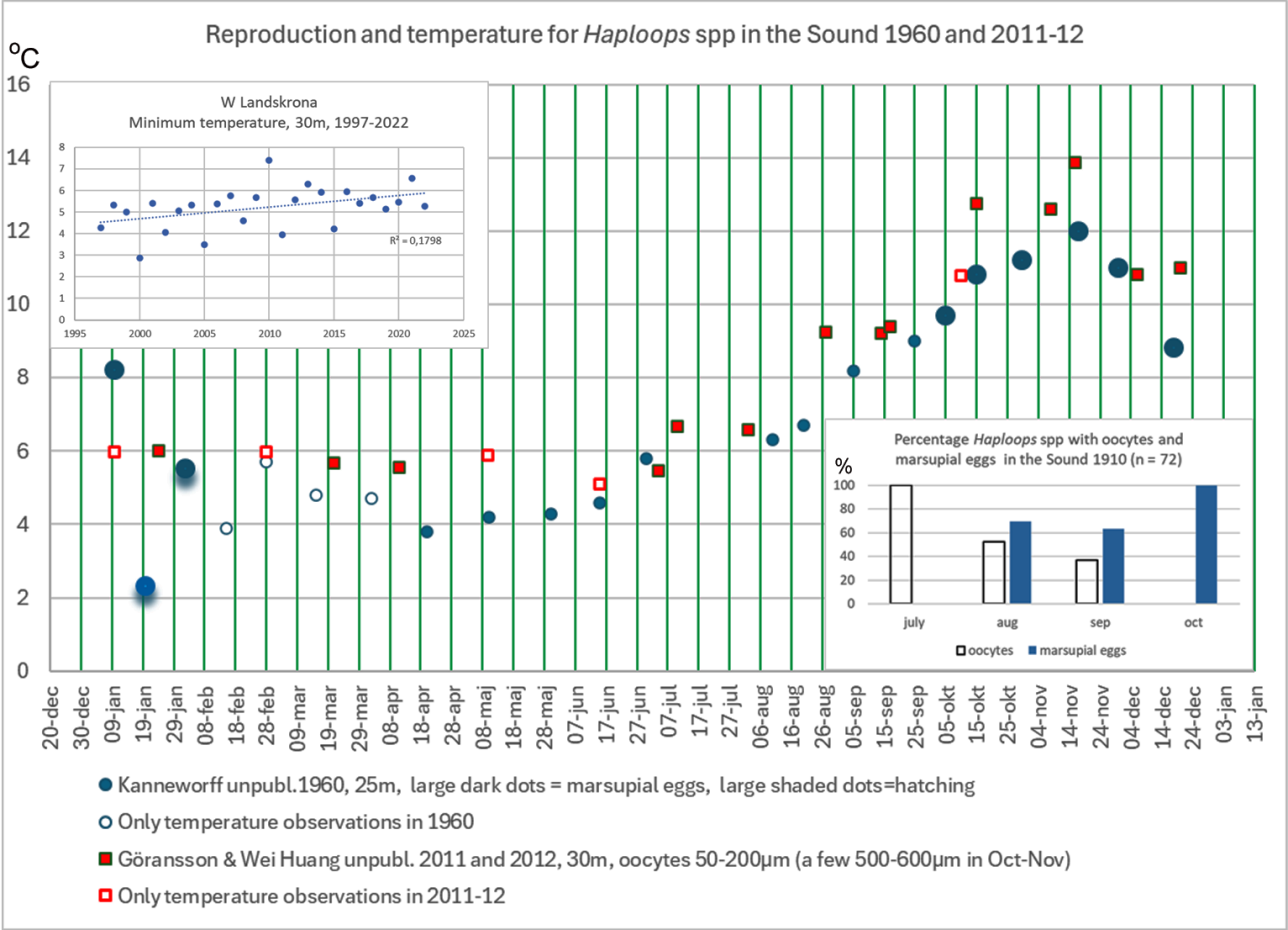
Temperature and reproduction in amphipods of genus *Haploops* in the Sound 1960 compared to 2011 and 2012. Large dark blue dots show observations of females with marsupial eggs. Large light blue dots show hatching. Small dots show observations of oocytes. Unfilled dots show temperature data only. Redrawn from Kanneworff (1966) and Göransson & Wei Huang 2011 and 2012 (*n* = 127/190). Inserted right lower corner: Percentage oocytes and marsupial eggs in amphipods of genus *Haploops* in different months in the Sound 1910. Amphipods collected by Björck (1915) and stored at the Zoological Museum, Lund University. Inserted left upper corner: Temperature at 30 m depth in the Sound 1997–2022. Linear regression, *r*2 = 0.1807, *p* = 0.03.

After the dramatic decline of the *Haploops* communities in the Sound in 2007, a follow up study of the remaining scarce population was carried out in 2011 and 2012. Almost no or very limited growth of oocytes (50–200 µm) was recorded for the two species, *H. tubicola* and *H. tenuis* and no oocytes did develop into mature eggs. Furthermore, none of the females carried eggs in the marsupium in the 16-month study, therefore, no hatching could be observed. The latter indicates very low or no recruitment at all in the Sound 2011–12. A comparison of the measured temperatures in the bottom water of the Sound shows generally higher temperatures in recent years compared to 1960, especially concerning the recorded lowest and highest temperatures (Fig. 12. Data from Kanneworff 1960 unpublished data and Göransson & Wei Huang, 2025, unpublished data.). Sjöstedt (1936) reported 4.3 °C as the minimum temperature and 10.5 °C as the maximum temperature for 30 m depth in the Sound in 1931. Compared to 2011 and 2012, this is approximately 2 to 3.5 °C degrees lower. This is a relatively high difference which also could lead to ecological consequences.

A review of *Haploops* material 1905–1937, stored at the Zoological Museum, Lund University of Sweden, confirmed Kanneworff’s data on the development of the marsupial eggs (Fig. 12 inserted). Oocytes dominated in July and August in 1910, and marsupial eggs were found from August and dominated from October (*n* = 72). Marsupial eggs also dominated all specimens from October 1913 (*n* = 110). This is an indication of a highly coordinated reproduction over time, which can be significant for the survival of this species.

Hypothetically, temperature can be of great importance for the reproduction of *Haploops*. Minimum temperatures at 30 m 1997–2022 increased from about 4.6 °C in 1997 to near 6 °C in 2022, *r*^2^ = 0.182, *p* = 0.03. In 2010 the minimum recorded temperature was 7.41 °C which could be well above temperature for the maturation of gonads. High temperature often leads to an increase in metabolism, which in a food shortage could lead to starvation when the gonads are being used as a metabolic substrate (Brockington & Clarke, 2001). The warmer climate may deeply impact amphipods with low temperature tolerances (Ritter & Bourne, 2024), but several other factors can adversely affect reproduction in amphipods (Hyne, 2011). However, it is not likely that pollution has affected the populations in the Sound because the load has generally decreased.

### Temperature and climate change

Increase of bottom temperature during 1997–2022 at the 15 to 30-meter level was on average 0.93 °C which is on the same level as for southern Kattegat (1.05 °C) 1993–2016, (Göransson, 2017a) and 0.18–0.31 °C per decade for the North American continental shelf 1968–2018 (Friedland et al., 2020). The increase is relatively high in the bottom water where the fauna in the Sound normally experiences temperatures in the range 5–12 degrees. In the Sound the reductions of the fauna were also the highest in deep water, well below the halocline, with narrow temperature variation.

However, it is not likely that the average temperature was the most important impact factor. Rather, periods of extreme temperatures may be more important, affecting, for example, reproduction and metabolism (Eriksson-Wiklund & Sundelin, 2001; Buckley & Huey, 2016; Kuan et al., 2022; Dougherty et al., 2024).

High temperatures in the bottom water are unusual in the Sound with its narrow temperature range.

Regarding low temperatures, there was a trend towards lower temperatures from the west coast and towards the colder Baltic Sea. Few low bottom water temperatures were recorded in the northern part of the west coast, while the conditions are relatively similar in the southern Kattegat and the Sound at a depth of 30 m. The lower temperatures and very narrow temperature range of the deep water of the Sound compared to the west coast are significant and already observed by Brattström (1941). Sjöstedt (1936) reports 4.3–10.5 °C at 30 m depth in 1931. These conditions are probably of great importance for benthic animals with different requirements in terms of metabolism and reproduction. On the other hand, species adapted to a narrow range of temperatures, like the conditions in the Sound, can be particularly vulnerable to relatively small changes. In the Sound it is probably these relatively small differences compared to the surrounding areas that make the fauna unique here with its northern touch. It also means that small changes can have major consequences. The temperature at 30 m depth in the Sound shows a slightly increasing trend during the period 1997–2022 (Kendall’s tau = 0.072, *p* = 0.015, *n* = 554). However, it is primarily the increase in low temperature readings that is striking with an increase of 1.33 °C in 25 years (Fig. 12). This is the highest increase in minimum temperature compared to the other four stations in this study. Besides the Sound, only F9 in the Bothnian Bay shows a significant increase in minimum temperature, with an increase of 0.83 °C in 25 years. In the Sound, the lowest temperatures were for the first time closer to 6 °C in 2007, in 2010 closer to 8 °C and after 2015 only temperatures above 5 °C have been measured. It is possible that these relatively high temperatures can significantly impact metabolism and reproduction. Warm winter temperatures may for instance lead to increased mortality and predation risk (Beardsley Christensen et al., 2023).

### Submergence and changed distribution of species

Total data for the Swedish coast concerning depth distribution for the ostracode *Philomedes brenda* and the bivalve *Nuculana pernula* shows roughly the same long-term change. The declining trends at lower depth intervals and increasing trends at deeper intervals can be interpreted as a result of an increasingly warmer climate. This response, that a species moves its range to greater depths when the temperature becomes too high in shallower water, is known as submergence (Haecker, 1904; Ekman, 1953). Regarding *P. brenda* is the Sound situated right on the south border of its distribution area (Kornicker, 1982). This Arctic-Boreal species, which earlier was common in the Kattegat, has also deceased sharply in this area (Göransson, 2017a).

A rich *Haploops*-population with a very narrow distribution still exists southeast of the island Läsö in the northern Kattegat. It is situated in the deepest part of the area, the Kattegat trench, at a depth of about 80 m (OCEANA, 2013). Initially, more than 8,000 ind/m^2^ were found in one sample (Hansen, 2020). In a follow-up survey in 2016, approximately 2,400 ind/m^2^ were recorded as the average of six grab samples (Pedersen & Deding, 2017). The annual bottom temperature varied approximately between 4.5 °C and 9.0 °C in this area (Nordberg, Filipsson & Malmgren, 1999). This can be compared with the populations in the Sound and southern Kattegat which lived at about 30 m depth at 4–14 °C (Fig. 12). According to Enequist (1949) the temperature range in the populations in the Skagerrak at 60–70 m depth was 4.3–11 °C and at 100 m about 5–8 °C. It may therefore be possible to speculate if the recent population at Läsö is another example of submergence, where species can distribute to deeper areas as the climate becomes warmer.

The differences between the changes in the Sound and the significantly colder Baltic Sea are much greater compared to the west coast. The amphipod *Pontoporeia femorata* and the priapulid *Halicryptus spinulosus* that decreased in abundance in the Sound showed no significant change in the Baltic Sea during this study period. It is of great value to compare the distributional pattern of the glacial relic *P. femorata* in ca 1935 (Ekman, 1953) with the records from studies 1998 and 2024 from the Swedish Species Information Centre (SLU, 2025). This comparison indicate that this cold-water species has disappeared from the relatively warm Swedish west coast, but has, on the other hand, expanded strongly northwards in colder parts of the Baltic Sea. The reduction in the Sound may thus be part of its retreat on the Swedish west coast due to a warmer climate.

### Transitions at range edges

The decline of several northern species in the Sound may be caused by the fact that they lived close to their southern distributional boundary or trailing range edge. This applies to both salinity-demanding species that live along the west coast and brackish water species that are typical of the Baltic Sea. The transition from a benthic community dominated by *Haploops* spp. to *Amphiura filiformis* at range edges can be an example of regime shift in response to global change (Hughes et al., 2017; Angeler et al., 2019). It is important that these results are interpreted considering how contemporary conditions deviate from historical selection regimes (Angert, Bontrager & Agren, 2020).

Populations occurring at range edges can be locally adapted to unique environmental conditions and have higher severity (Dawson et al., 2011) and frequency of extreme climatic events relative to the range core. Therefore, range edge genotypes are better adapted to extreme climates relative to core populations (Rehm et al., 2015). Phenotypic plasticity also enables rapid responses to environmental change and could facilitate range shifts in response to climate change. What drives the evolution of plasticity at range edges, and the capacity of range-edge individuals to be plastic, remain unclear (Usui et al., 2023). The reason the isolated *Haploops*-community was able to remain in the Sound until recently may be due to the above factors. Despite this, they have disappeared, probably due to the synergistic impact of climate change and hypoxia. However, the risk of extinction in isolated populations with extremely low connectivity (Bahn, O’Connor & Krohn, 2006) should also be considered.

Many salinity-demanding species have their range edge in the Sound due to the strong encounter with the brackish Baltic Sea. The Limhamn/Drogden sill and the strong halocline create unusually strong hard edges and a rare dynamic hydrography that organisms must deal with. This creates a selection pressure that results in stress-resistant populations valuable to the local environment. Loss of these populations could be significant for the ability of species to persist in the face of rapid future climate change (Rehm et al., 2015).

The *Haploops*-community had structural characteristics that could be considered hard edge. This is in view of its clear demarcation with high densities against the surrounding *Amphiura*-community, which should instead be described as soft edge in view of its more gradual demarcation (Watts, Waldron & Kuchta, 2024). Trailing-edge species with harder range edges are slightly more likely to be declining. In contrast, soft-edge species with harder range edges are more likely to increase (Gilbert et al., 2024). While climate-driven extirpations have occurred in the ocean, these contractions appear to be five times slower than the rate of range expansions (Poloczanska et al., 2016). In addition, the proportion of leading range edges that are expanding is greater than the proportion of trailing edges that are contracting (Pinsky, Selden & Kitchel, 2020). In the Sound, with its limited size, there are only examples of contracting trailing edges of cold-water species. However, one of the these, the brackish water species *Pontoporeia femorata* has its trailing edge in the Sound and on the west coast, and its leading edge in the Baltic Sea. This cold-water species has declined in its trailing edge in the warmer Sound and seems to have increased its distribution in its leading edge northward in the cold Baltic Sea. This is a typical response to an increasingly warm climate (Pinsky, Selden & Kitchel, 2020).

## Conclusions

The major goal of this study was to provide a comparison between different areas based on results from the Sound. The selected temperature stations, only provides a rough estimate of the large differences between the areas and does not represent bottom-water conditions at all the benthic sampling sites. Additionally, the use of a nonparametric test primarily provides an idea of the direction of changes. Hopefully, this can increase interest in future more detailed studies.

The recent decline of many northern species in the Sound has a history that is documented far back in time, but sampling has varied regarding methods and continuity. Comparisons with other locations are also hampered by variations in depth, differences in sampling gear, uneven replication and several complicating factors. This is a great problem and further stresses the need to preserve long-term series of data collected at the same location and with the same methods.

The strong vertical stratification and regional differences in salinity and temperature across the Sound, Kattegat/Skagerrak, and the Baltic Sea also makes direct comparisons between the subareas difficult. The unique hydrographic conditions in this area are largely responsible for the differences in species composition (Ekman, 1953).

The importance of climate warming is hard to separate from other drivers, such as eutrophication, hypoxia, trawling, and predation, to explain observed faunal changes. All of these factors are of great importance and can interact, which to some extent is evident from the reported results. Temperature affects many processes, including production, oxygen conditions and predation. However, the trawl ban in the Sound excludes direct impact of trawling, especially of species with non-pelagic larvae. Predation from fish has probably not increased because stocks have actually declined. Nitrogen levels have decreased, which may result in reduced organic deposition. This can lead to a reduced number of species, especially species that seem to benefit from high organic load, *e.g.*, *Haploops* spp.

The development in individual abundance 1998–2022 in the Sound with its trawl-ban was similar to heavily trawled areas along the Swedish west coast. The fact that primarily northern species decreased and southern species increased points to the impact of climate change with increased temperature. This has previously been indicated in the Kattegat 1993–2016 (Göransson, 2017a) but has here been shown to be more evident in the Sound.

The bottom water temperature is of great importance for the distribution of species (Künitzer, 1992; Reiss et al., 2011; Friedland et al., 2020).

Indications of submergence and disturbed reproduction may be additional signs of impacts of increased temperature.

The northern species of the Sound, at their limit of distribution, most likely have a low adaptive capacity to climate change. The fact that the changes affected common and typical species for the Sound should be put in relation to Gunnar Thorson’s consideration that benthic faunal communities were ”distinct and constant”, and that Petersen’s communities were the same after 40 years (Thorson, 1957). The changes should therefore be taken seriously, as the faunal composition in the Sound previously has been relatively stable despite very large variations in hydrography.

Increased temperature can be an explanation why southern species with pelagic larvae have increased in recent times. Sea temperatures which control the ripening of gonads and spawning are significant as limiting factors for the distribution of adult invertebrates. Non pelagic larvae are probably, inter alia, an adaptation to low temperature habitats according to Thorson’s rule. The deep benthic fauna of the Sound appears particularly vulnerable to climate change since many species here live very close to their distributional boundary.

However, several factors other than climate change may have contributed to the observed faunal changes. It is very likely that bottom trawling in the adjacent Kattegat has an impact on the presence of many benthic animals in the Sound by reducing the import of pelagic larvae. But these species have a much larger catchment area for larvae than species with reproduction connected to the bottom.

The results also indicate that the changes are highest on the deepest bottoms. This is probably because the relative change in temperature is highest on bottoms with a narrow temperature range. This is characteristic of the deep water in the Sound. The ocean has also become significantly more stratified over the last half century as the climate has warmed (Li et al., 2020) which also has been recorded regionally in the Baltic Sea in a 35-year perspective (Taavi & Urmas, 2019). This is especially alarming for the environment in the strongly stratified Sound and could have extensive consequences for the entire ecosystem. A stronger stratification can, for instance, lead to poorer oxygen conditions in the bottom water.

Hypothetically, the hypoxia in 2007 combined with high temperatures could likely be the factor that started the decline of *Haploops* in the Sound. Subsequently, the high temperatures may adversely affect reproduction and weaken the isolated population to a level too low to survive.

The transition from a *Haploops* community to an *Amphiura* community roughly means increased bioturbation in terms of dominant species (Queirós et al., 2013; Weinert et al., 2022). However, the loss of a habitat (community), implies a reduction of the total regional biological variation in the area (gamma diversity). The nutritional value, as commercial fish food considered, should be significantly higher long-term for the crustacean-dominated *Haploops* community compared to the echinoderm dominated *Amphiura*-community. In the Sound, *Haploops* spp., *Pontoporeia femorata* and other amphipods are important food sources especially for young cod *Gadus morhua*. Amphipods have a relatively high nutrient and fat content and are therefore a valuable food source for many organisms.

It seems that the deep bottoms of the Sound have lost some of their “secret and richness in the depths” (Thorson, 1950b).

### The Sound as an important protected reference area

It is very important to continue the history of environmental data in the trawl free Sound for long term ecological and environmental studies. The Sound is a sensitive area with a low adaptive capacity to temperature changes since many species live near the edge of their geographical distribution. Therefore, further large-scale changes will probably be detected at an early stage. However, it is important to protect the Sound as a relatively undisturbed, trawl-free, reference area for future studies of changes in the marine environment. It is also important to continue and preserve a large number of ecosystem services, not only on energy and matter cycles, but also on essential habitats. The small and scattered areas that currently are protected are probably not sufficient to maintain the diversity in the area. The trawling ban should be biologically justified, and not just due to heavy vessel traffic. The relatively unaffected bottom environment should also be protected against other significant impacts. Protective regulations for the shared waterway need to be coordinated between the two neighboring countries. This is strong indications that a joint Danish Swedish protection of the entire Sound is needed, preferably in the form of a national park. This could be connected to already protected areas in the southern Kattegat to create a unique large trawl-free area between the Atlantic and the Baltic Sea.

##  Supplemental Information

10.7717/peerj.20996/supp-1 Supplemental Information 1 Sampling stations Positions and depths for sampling stations

## Additional Information and Declarations

### Data Availability

The following information was supplied regarding data availability: The data is available in the Supplemental Files and at https://www.smhi.se/data/hav-och-havsmiljo/datavardskap-oceanografi-och-marinbiologi using search parameters: zoobenthos, species, station names, years.
